# Carotid Subclavian Bypass for the Treatment of Coronary Subclavian Steal Syndrome

**DOI:** 10.21470/1678-9741-2020-0609

**Published:** 2022

**Authors:** Lucas Yuji Sonoda, Maria da Graça Lepre Hawerroth, Túlio Torres Vargas, Luciano Batista Silveira Santos, Thomas Rezende Diniz, Walter Alvarenga de Oliveira, Maria Ambrosina Cardoso Maia

**Affiliations:** 1 Minas Gerais State University, Passos, Minas Gerais, Brazil.

**Keywords:** Coronary Subclavian Steal Syndrome, Subclavian Artery, Surgical Procedure, Operative, Peripheral Arterial Disease

## Abstract

Coronary subclavian steal syndrome is an uncommon cause of angina in patients with a previous coronary artery bypass graft procedure. The patient had chest pain with the exertion of the left upper limb, difference in blood pressure between the left and right arm, occlusion at the ostium of the left subclavian artery. He underwent carotid subclavian bypass surgery that was successful in relieving symptoms. On the other hand, the patient had an embolic stroke related to the procedure and further assessment may be necessary.

**Table:** Abbreviations, acronyms & symbols

CABG	= Coronary artery bypass graft
CSSS	= Coronary subclavian steal syndrome
LAD	= Left anterior descending artery
LIMA	= Left internal mammary artery
LSA	= Left subclavian artery
MI	= Myocardial infarction

## INTRODUCTION

The coronary subclavian steal syndrome (CSSS) is an uncommon cause of angina in patients with chronic coronary artery disease. It occurs with the exertion of the left upper limb in patients who underwent coronary artery bypass graft (CABG) surgery using the left internal mammary artery (LIMA) and severe stenosis (>75%) of the left subclavian artery (LSA) before the origin of LIMA. The blood flow is “stolen” from the coronary circulation towards the LSA distally, which can precipitate stable angina, acute coronary syndrome, or cardiac arrhythmias^[1]^.

### Case Presentation

A 62-year-old man with a history of hypertension, smoking, and coronary heart disease was admitted to the cardiology service with typical chest pain that worsened with left upper limb physical activities and relieved at rest, limiting him to perform daily activities (typical angina Canadian Cardiovascular Society [CCS] III).

**Fig. 1:**
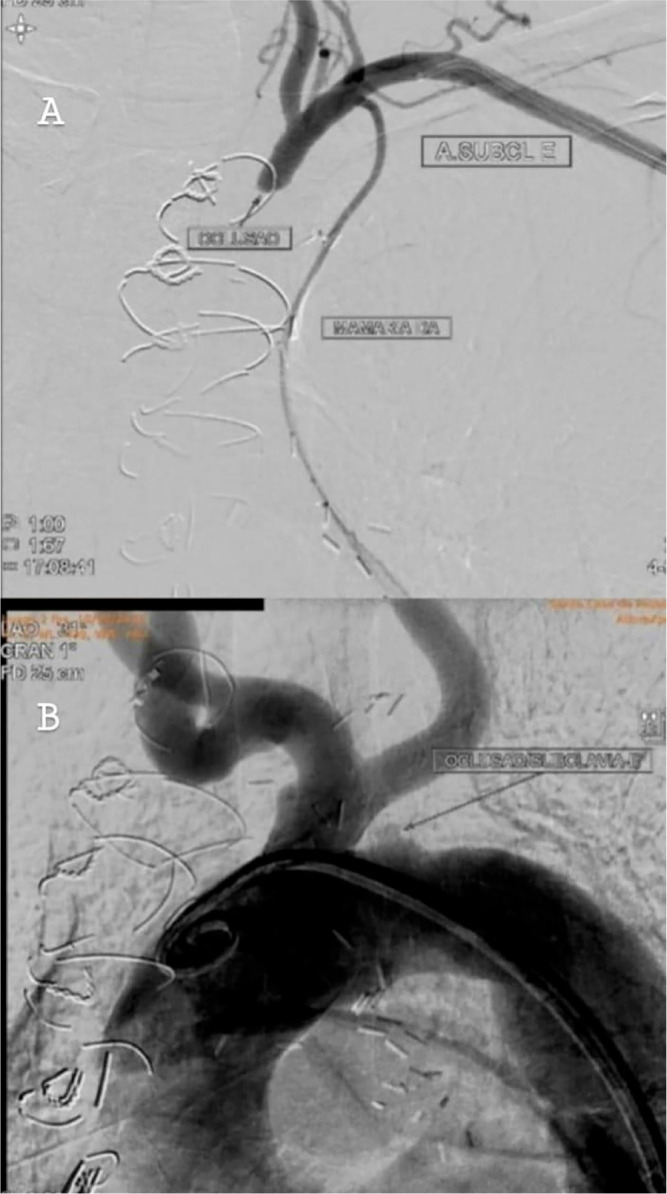
(A) Angiography of left vertebral artery and LIMA showing occlusion of the LSA. (B) Angiography of the aortic arch showing occlusion of the LSA. LSA = left subclavian artery

When this patient was 53 years old, he had an acute myocardial infarction (MI) in the inferior wall and was diagnosed with many severe coronary obstructions with a three-vessel disease pattern. He underwent CABG with the following vessel revascularization: radial artery graft from the aorta to the first diagonal artery, saphenous vein graft from the aorta to the first marginal artery, saphenous vein graft from the aorta to the posterior descending artery of the right coronary artery, and LIMA graft to the left anterior descending artery (LAD). Lately, the patient suffered two non-ST elevation MI with percutaneous revascularization. A supra-aortic vessel angiography was performed, which showed occlusion of the LSA, as presented in Figure 1.

On physical examination, the patient showed differences in blood pressure between the right upper limb (180/100 mmHg) and the left upper limb (120/80 mmHg). The electrocardiogram showed Q waves in inferior leads due to the previous MI. Echocardiogram revealed preserved left ventricular ejection fraction. Laboratory tests were within normal values, despite LDL-cholesterol above the target for secondary prevention because of irregular medication intake.

His prescription was adjusted as follows: metoprolol succinate 50 mg per day, losartan 100 mg per day, hydrochlorothiazide 12.5 mg per day, rosuvastatin 20 mg per day, aspirin 100 mg per day, clopidogrel 75 mg per day and spironolactone 25 per day.

**Fig. 2:**
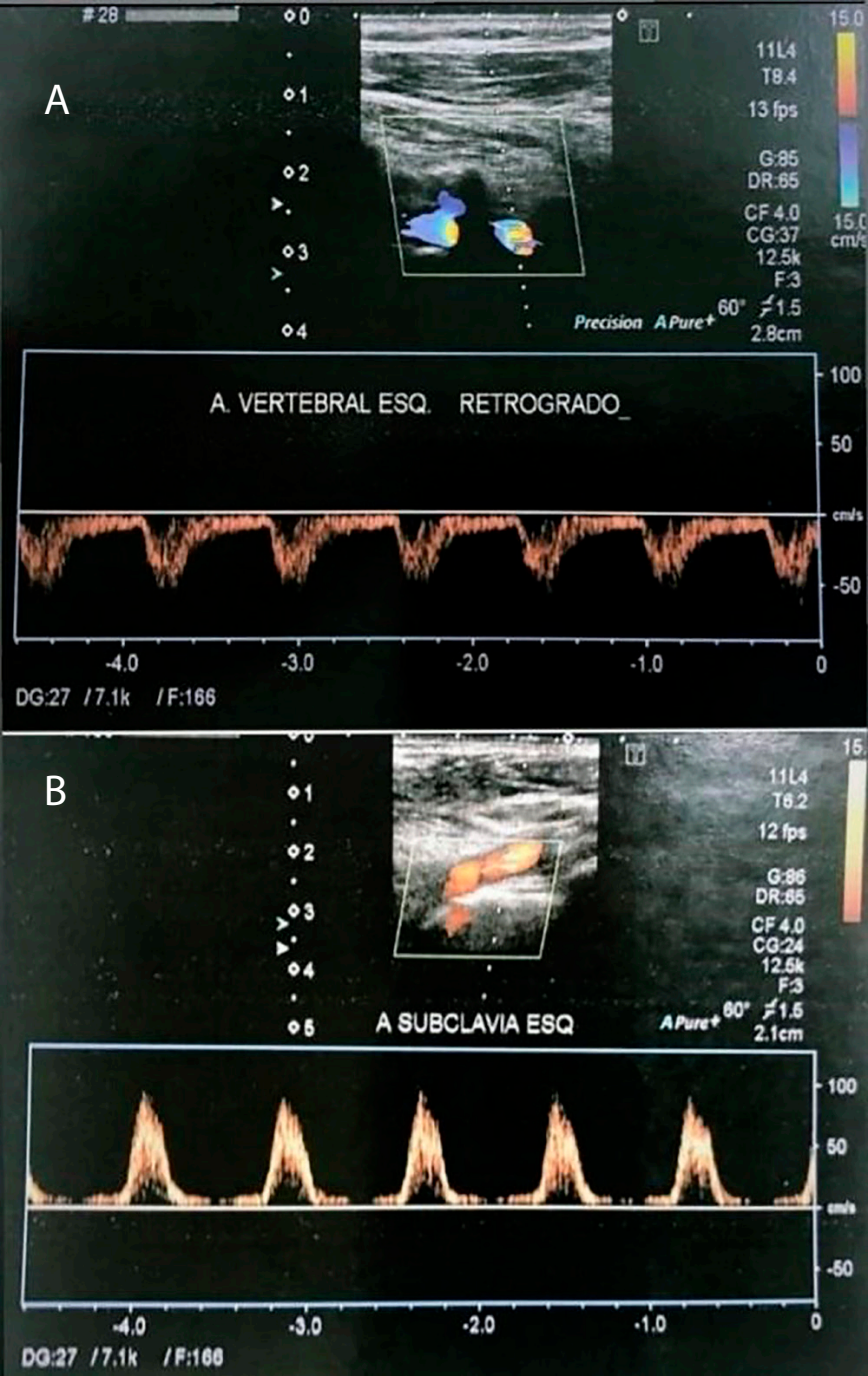
(A) Doppler ultrasonography of the left vertebral artery showing retrograde flow. (B) Doppler ultrasonography of the LSA revealing proximal occlusion and distal flow with a postobstructive biphasic spectral pattern.

A Doppler ultrasonography of the carotid, vertebral and subclavian arteries was performed, which demonstrated: anterograde flow in the right vertebral artery and completely reversed flow in the left vertebral artery (Figure 2), denoting complete/permanent type 3 steal^[2]^; proximal occlusion of the LSA, in addition to multiple obstructions without hemodynamic repercussions. The left common carotid artery had no lesions.

After 8 months of clinical management and good adherence to medications, he still had angina on exertion. Calcium channel blockers and nitrate were introduced, without remission of symptoms after reevaluation in 14 days.

### Technical Description

**Fig. 3:**
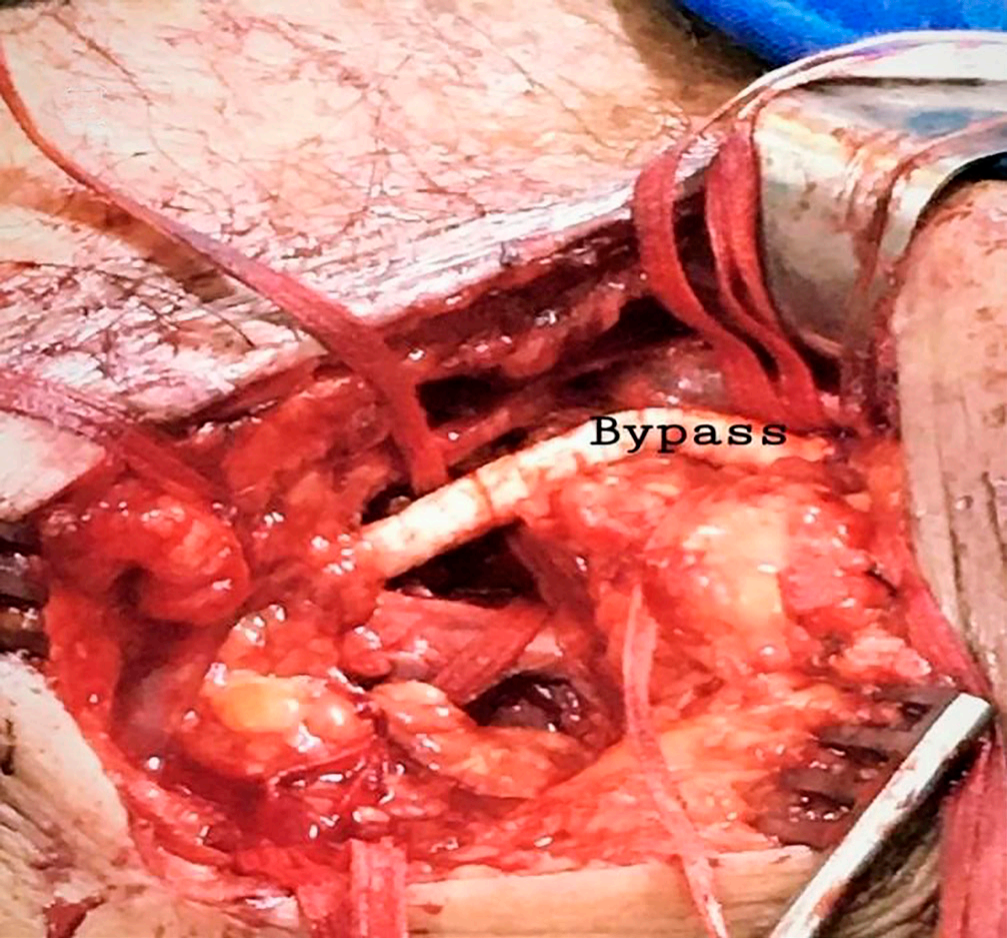
Intraoperative image after coronary subclavian bypass.

This case was discussed with a vascular surgeon and interventional cardiologist. A carotid-subclavian bypass with a Dacron graft was indicated (Figure 3). Endovascular intervention was contraindicated considering the risk of aortic dissection with retrograde recanalization—there was occlusion right at the LSA ostium.

During surgery, the patient remained hemodynamically stable, the left common carotid was clamped for 16 minutes and there were no complications.

Postoperatively, the patient recovered well, with complete remission of anginal symptoms. However, on the 3^rd^ postoperative day, he presented left homonymous hemianopsia secondary to embolic stroke in the right occipital region. A non-contrast-enhanced cranial tomography showed a hypodense area with partially defined limits, with a cortico-subcortical location in the right occipital area. He was reevaluated after 14 days of surgery and maintained remission of anginal symptoms. The difference in blood pressure between the upper limbs decreased (150/80 in the right upper limb and 120/80 in the left upper limb). Doppler ultrasonography showed anterograde flow in the LSA. However, the confrontation visual field test showed homonymous hemianopsia with slight adaptation.

## COMMENTS

CSSS caused by LSA stenosis is considered an unusual repercussion of CABG using LIMA, and the prevalence ranges from 0.2 to 6.8%^[1]^.

About 90% of subclavian artery obstructions occur due to atherosclerosis. Other causes are arteritis, inflammation, radiation, neurofibromatosis, fibromuscular dysplasia, and compressive syndromes^[1]^.

The diagnosis of LSA stenosis is suspected when there is a significant difference in blood pressure between the upper limbs (≥15%). The gold standard test is subclavian angiography. Alternatives are Doppler ultrasonography, computed tomography, and magnetic resonance^[1,3]^.

Subclavian revascularization is indicated when there is angina refractory to optimized clinical management, acute coronary syndromes, ventricular arrhythmia, or decompensated heart failure^[1]^. Angioplasty with percutaneous stenting is a good treatment option with a high success rate^[4]^.Surgical bypass procedure is considered when endovascular treatment cannot be achieved or fails and in symptomatic patients with low operative risk. Bypass can be performed through the carotid-subclavian shunt; in addition to anastomoses in other extrathoracic vessels, it has shown good results with remission of symptoms^[1,3,5-7]^.

Thus, in this case a patient with CSSS underwent carotid-subclavian bypass surgery due to the ineffectiveness of optimized clinical treatment and the risk of aortic dissection with percutaneous retrograde LSA recanalization. The procedure was successful in achieving remission of angina, but it was complicated by an embolic ischemic stroke in the right occipital region on the 3^rd^ postoperative day.

### Research with Human Subjects and Experimental Studies

This research was conducted with the informed and appropriate consent of the participant. It was approved by the Ethics Committee of the Santa Casa de Misericórdia de Passos. Approval number: 4.006.584.
